# Association between the time-varying arterial carbon dioxide pressure and 28-day mortality in mechanically ventilated patients with acute respiratory distress syndrome

**DOI:** 10.1186/s12890-023-02431-6

**Published:** 2023-04-19

**Authors:** Rui Zhang, Hui Chen, Ran Teng, Zuxian Li, Yi Yang, Haibo Qiu, Ling Liu

**Affiliations:** 1 Jiangsu Provincial Key Laboratory of Critical Care Medicine, Department of Critical Care Medicine, School of Medicine, Zhongda Hospital, Southeast University, Nanjing, Jiangsu 210009 China; 2 Department of Critical Care Medicine, The First Affiliated Hospital of Soochow University, Suzhou, Jiangsu 215000 China

**Keywords:** Acute respiratory distress syndrome, Mechanical ventilation, Arterial carbon dioxide pressure, 28-day mortality

## Abstract

**Background:**

Recent studies have shown an association between baseline arterial carbon dioxide pressure (PaCO_2_) and outcomes in patients with acute respiratory distress syndrome (ARDS). However, PaCO_2_ probably varies throughout the disease, and few studies have assessed the effect of longitudinal PaCO_2_ on prognosis. We thus aimed to investigate the association between time-varying PaCO_2_ and 28-day mortality in mechanically ventilated ARDS patients.

**Methods:**

In this retrospective study, we included all adult (≥ 18 years) patients diagnosed with ARDS who received mechanical ventilation for at least 24 h at a tertiary teaching hospital between January 2014 and March 2021. Patients were excluded if they received extracorporeal membrane oxygenation (ECMO). Demographic data, respiratory variables, and daily PaCO_2_ were extracted. The primary outcome was 28-day mortality. Time-varying Cox models were used to estimate the association between longitudinal PaCO_2_ measurements and 28-day mortality.

**Results:**

A total of 709 patients were eligible for inclusion in the final cohort, with an average age of 65 years, of whom 70.7% were male, and the overall 28-day mortality was 35.5%. After adjustment for baseline confounders, including age and severity of disease, a significant increase in the hazard of death was found to be associated with both time-varying PaCO_2_ (HR 1.07, 95% CI 1.03–1.11, p<0.001) and the time-varying coefficient of variation for PaCO_2_ (HR 1.24 per 10% increase, 95% CI 1.10–1.40, p<0.001) during the first five days of invasive mechanical ventilation. The cumulative proportion of exposure to normal PaCO_2_ (HR 0.72 per 10% increase, 95% CI 0.58–0.89, p = 0.002) was associated with 28-day mortality.

**Conclusion:**

PaCO_2_ should be closely monitored in mechanically ventilated ARDS patients. The association between PaCO_2_ and 28-day mortality persisted over time. Increased cumulative exposure to normal PaCO_2_ was associated with a decreased risk of death.

**Supplementary Information:**

The online version contains supplementary material available at 10.1186/s12890-023-02431-6.

## Background

Acute respiratory distress syndrome (ARDS), characterized by refractory hypoxemia, is a life-threatening disease with high incidence and high mortality [1, 2]. More than 70% of ARDS patients require invasive mechanical ventilation [1–3] to maintain oxygenation and ventilation. However, arterial carbon dioxide pressure (PaCO_2_), a frequently monitored parameter that is closely related to alveolar ventilation [4], has not been fully appreciated and emphasized in clinical studies. Indeed, PaCO_2_ derangements, including hypercapnia and hypocapnia, are pretty prevalent in ARDS patients [5], and numerous studies evaluating the association between PaCO_2_ and clinical outcomes have yielded different results [5–7]. To date, there are no definitive guidelines on how to manage PaCO_2_ in patients with ARDS.

However, published studies investigating the impact of PaCO_2_ on the prognosis of ARDS patients have merely focused on a single measurement, typically within 24 or 48 h after admission to the intensive care unit (ICU) or mechanical ventilation. Statistical analyses that only adjust for baseline confounders seem to be implausible, as PaCO_2_ possibly varies due to the evolution of the disease and the corresponding therapeutic regimens. Static assessments of PaCO_2,_ which ignored the dynamic nature of the syndrome, may be incomprehensive and would fail to consider the relation of survival as a function of the change in the covariate [8]. In fact, compared with analyses based exclusively on cross-sectional data, the analysis of longitudinal data can sometimes yield additional or even different information to guide clinical decisions [9–11]. It is still unclear whether the association between time-varying PaCO_2_ and mortality is significant and remains persistent over time.

Therefore, we proposed to evaluate the effect of dynamic PaCO_2_ after the initiation of mechanical ventilation, as measured by either the time-varying daily PaCO_2_ or the coefficient of variation for PaCO_2_, on 28-day mortality in patients with acute respiratory distress syndrome. We also examined whether there was a cumulative effect of exposure to PaCO_2_ derangement over time.

## Methods

### Study design and patients

In this retrospective study, data were collected from the Department of Critical Care Medicine, Zhongda Hospital, Southeast University. Between January 2014 and March 2021, patients with ARDS meeting the Berlin definition [12] who were admitted to the ICU were screened. Eligible patients were 18 years of age or older and received invasive ventilation for at least 24 h. Patients who only received non-invasive ventilation support were excluded. Since ECMO can dramatically affect PaCO_2_ independent of other respiratory support [13], we excluded that subset of patients. The Research Ethics Commission of Zhongda Hospital approved the present study (approval number: 2022ZDSYLL279-P01) and waived the written informed consent since deidentified data were extracted from the medical record, and the personal information was kept confidential.

### Data collection and outcomes

Demographic data, comorbidities, baseline severity scores, clinical outcomes, and laboratory tests were retrospectively collected. Variables at the initiation of mechanical ventilation were defined as the baseline. All results of arterial blood gas analysis during the first five days of invasive ventilation were extracted. The following definitions were applied: hypocapnia (PaCO_2_ < 35 mmHg), normocapnia (35 mmHg ≤ PaCO_2_ ≤ 45 mmHg), and hypercapnia (PaCO_2_ > 45 mmHg). Ventilator parameters, including tidal volume, respiratory rate, positive end-expiratory pressure (PEEP), plateau pressure (Pplat), and peak inspiratory pressure (Ppeak), were monitored and recorded hourly. The weighted average of each parameter was calculated every 8 h, referring to the previous literature [9]. The dynamic driving pressure, mechanical power, and ventilatory ratio were calculated. Therapeutic strategies were collected, including prone position, recruitment maneuvers, neuromuscular blockers, and vasopressors, from Day 1 to Day 5 since mechanical ventilation.

The primary outcome was overall 28-day mortality. Secondary outcomes were ICU and in-hospital mortality and ventilator-free days (VFDs) over 28 days. Patients who died before Day 28 were considered to have zero VFDs. The details of data collection and definition are presented in the supplement.

### Statistical analysis

Categorical variables are reported as counts (proportions) and were compared with the chi-square or Fisher’s exact test. Continuous variables are presented as the mean (standard deviation, SD) or median [interquartile range (IQR)] and were compared with Student’s t-test or the Mann–Whitney U test as appropriate.

PaCO_2_ was measured repeatedly and changed over the follow-up period, which was considered a time-varying covariate. The coefficient of variation for PaCO_2_ (CV-PaCO_2_, defined as the percentage of standard deviation to the mean PaCO_2_ over a specific period), which could reflect the amplitude of change in PaCO_2_, was also considered a time-varying covariate. Respiratory variables which can influence CO_2_ elimination [14] and well-established markers of lung injury, such as driving pressure and mechanical power [15, 16] were included as time-varying covariates. To assess the impact of time-dependent PaCO_2_ exposure on 28-day mortality, the time-dependent Cox model was implemented to adjust for both time-fixed and time-varying covariates [8]. According to previous studies, we adjusted for the severity of illness by adding age, Acute Physiology and Chronic Health Evaluation (APACHE) II score, and PaO_2_/FiO_2_ ratio at baseline. Prespecified subgroup analyses were used to investigate whether the effects of time-varying PaCO_2_ differ in patients categorized by severity, etiology, ventilatory strategies, and ventilation impairment.

We also investigated the association between the cumulative effect of PaCO_2_ and 28-day mortality. The cumulative exposure of PaCO_2_ was quantified by the proportion of tests suggestive of normocapnia to the total number of arterial blood gas analyses. The adjusted association between potentially harmful exposure (PaCO_2_ derangements) and mortality was depicted by Loess smoothing analysis. The number of missing or censoring values is presented in Table S1. Variables with a missing ratio of more than 25% were excluded from the final analysis. Outliers were censored, and missing values of less than 25% were replaced using multiple imputations by chained equations. The statistical analyses were conducted using R version 4.0.2. The level of significance was set at 0.05 (two-tailed).

## Results

### Patient characteristics

**Fig. 1:**
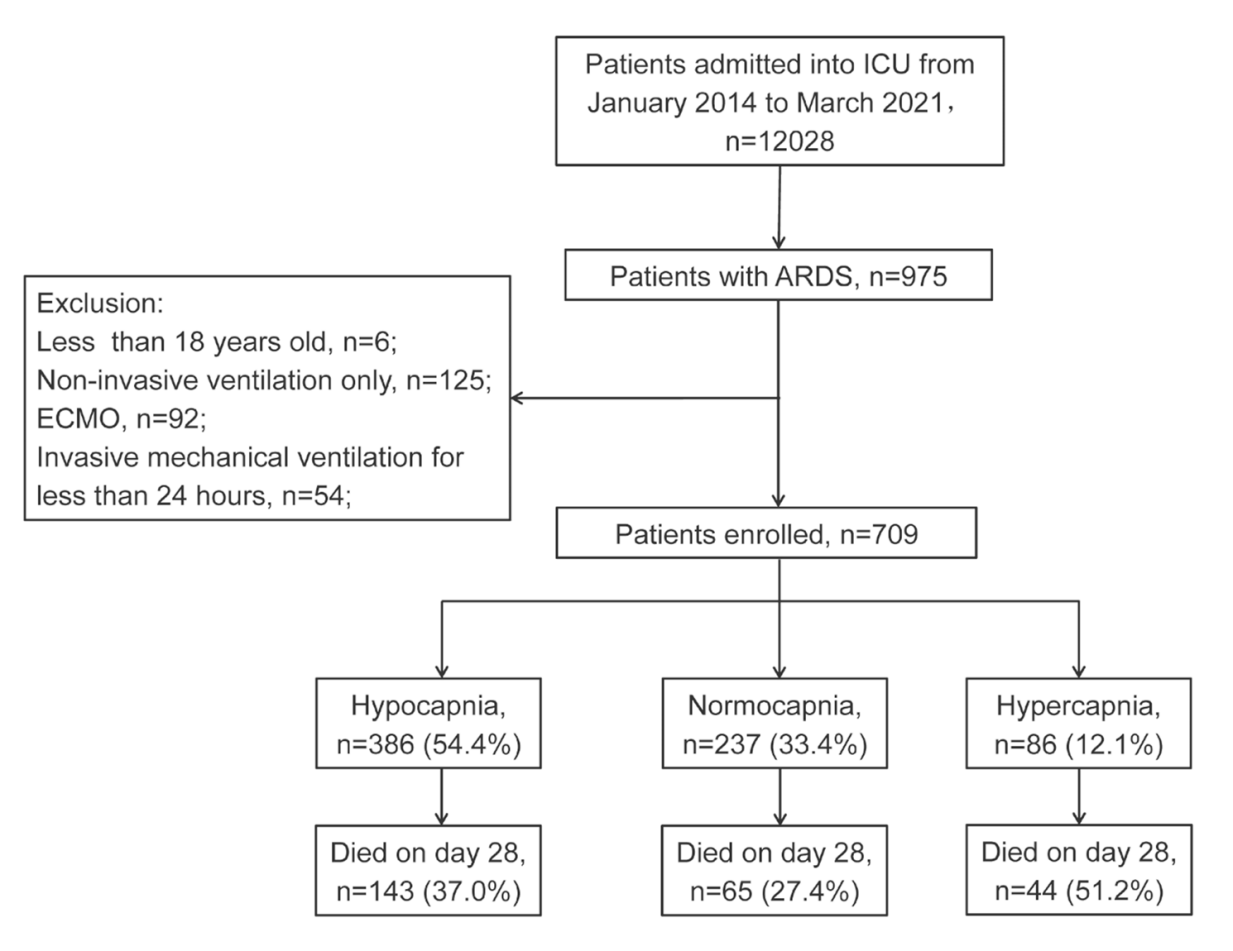
Flowchart of patient enrolment

A total of 975 ARDS patients were screened, and we identified 709 patients (Fig. 1) in the final cohort with a total of 10,883 PaCO_2_ measurements. By Day 5 sicne commencement of mechanical ventilation, 663 patients survived, 474 of whom were still mechanically ventilated. Most patients were men (70.7%), with a mean age of 65 (± 16) years. Pneumonia was the leading cause of lung injury, followed by non-pulmonary sepsis, and more than 80% of patients had a concurrent diagnosis of sepsis (Table 1). Patients exposed to hypocapnia, normocapnia, and hypercapnia at baseline accounted for 54.4%, 33.4%, and 12.1%, respectively.

**Table 1:** Demographic data and clinical outcomes of enrolled patients

	Overall (n = 709)	Survivors (n = 457)	Non-survivors (n = 252)	P values
Age (years)	65 (16)	63 (17)	69 (15)	< 0.001
Male, n (%)	501 (70.7)	331 (72.4)	170 (67.5)	0.192
Body weight(kg)	68.0 [60.0, 75.0]	70.0 [60.0, 75.0]	65.0 [58.8, 70.0]	< 0.001
BMI	23.6 (3.9)	24.1 (4.2)	22.8 (3.3)	< 0.001
APACHE II	23 (8)	21 (8)	26 (7)	< 0.001
SOFA	9 (4)	8 (3)	10 (4)	< 0.001
Primary lung injury, n (%)				0.098
Pneumonia	453 (63.9)	279 (61.1)	174 (69.0)	
Non-pulmonary sepsis	120 (16.9)	78 (17.1)	42 (16.7)	
Aspiration	54 (7.6)	37 (8.1)	17 (6.7)	
Trauma	51 (7.2)	38 (8.3)	13 (5.2)	
Other	31 (4.4)	25 (5.5)	6 (2.4)	
Pulmonary ARDS, n (%)	547 (77.2)	347 (75.9)	200 (79.4)	0.342
Sepsis, n (%)	607 (85.6)	377 (82.5)	230 (91.3)	0.002
Comorbidities, n (%)				
Hypertension	354 (49.9)	220 (48.1)	134 (53.2)	0.228
Diabetes	168 (23.7)	102 (22.3)	66 (26.2)	0.286
Chronic heart disease	144 (20.3)	74 (16.2)	70 (27.8)	< 0.001
Chronic kidney disease	74 (10.4)	40 (8.8)	34 (13.5)	0.065
Pulmonary disease	45 (6.3)	25 (5.5)	20 (7.9)	0.259
Solid tumor	67 (9.4)	37 (8.1)	30 (11.9)	0.127
Clinical outcomes				
VFD 28 (day)	10 [0, 22]	21 [12, 24]	0 [0, 0]	< 0.001
ICU mortality, n (%)	184 (26.0)	14 (3.1)	170 (67.5)	< 0.001
ICU-LOS (day)	12 [7, 20]	15 [9, 25]	8 [4, 15]	< 0.001
Hospital mortality, n (%)	198 (27.9)	17 (3.7)	181 (71.8)	< 0.001
Hospital-LOS (day)	20 [11, 31]	26 [15, 37]	12 [6, 20]	< 0.001

APACHE II: Acute Physiology and Chronic Health Evaluation II; BMI: body mass index; ICU: intensive care unit; LOS: length of stay; VFD: ventilator-free day. Data are presented as mean (SD), median (interquartile range [IQR]), or counts (proportion)

The characteristics of respiratory and hemodynamic parameters at baseline are described in Table 2. The mean PaO_2_/FiO_2_ and PaCO_2_ were 202.2 (± 88.5) mmHg and 35.8 (± 12.3) mmHg, respectively. All patients received an average tidal volume of no more than 8 mL/kg predicted body weight (PBW). 18.1% of patients received prone position ventilation, and no more than 10% of patients received recruitment maneuvers or neuromuscular blockers. Compared with the survivors, non-survivors had a lower PaO_2_/FiO_2_ and pH, while dynamic driving pressure and mechanical power were significantly higher in the non-survivors. Baseline PaCO_2_ was comparable in both groups, and the ventilatory ratio was higher in the non-survivors, but the difference was not statistically significant. The survivors were more hemodynamically stabilized, as indicated by the higher mean arterial pressure, lower serum lactate, and fewer requirement of vasopressors. More details about the population are shown in Table S2.

**Table 2:** Respiratory and hemodynamic parameters of enrolled patients

	Overall (n = 709)	Survivors (n = 457)	Non-survivors (n = 252)	P values
Respiratory parameters				
pHa	7.37 (0.09)	7.38 (0.08)	7.36 (0.11)	< 0.001
PaCO_2_ (mmHg)	35.8 (12.3)	35.6 (11.2)	36.3 (14.2)	0.492
PaO_2_ (mmHg)	113.2 (67.1)	115.3 (68.8)	109.3 (64.1)	0.255
PaO_2_/FiO_2_ (mmHg)	202.2 (88.5)	208.6 (86.7)	190.5 (90.6)	0.009
Bicarbonate (mmol/L)	20.8 (5.0)	21.2 (5.0)	20.1 (5.1)	0.003
Respiratory rate (bpm)	24 (8)	24 (8)	25 (7)	0.052
TV (mL/kg PBW)	7.0 (1.4)	7.0 (1.3)	7.0 (1.4)	0.748
min ventilation (L)	8.3 (2.3)	8.2 (2.3)	8.5 (2.1)	0.101
PEEP (cmH_2_O)	8 [5, 10]	8 [5, 10]	8 [7, 10]	0.001
Ppeak (cmH_2_O)	22.7 (5.7)	22.0 (4.6)	24.0 (7.1)	< 0.001
Dynamic DP (cmH_2_O)	14.3 (4.9)	14.0 (4.4)	14.9 (5.7)	0.028
Dynamic Crs (mL/cmH_2_O)	33.4 (13.6)	34.0 (13.1)	32.3 (14.4)	0.177
Ventilatory ratio	1.3 (0.6)	1.2 (0.5)	1.3 (0.6)	0.050
Mechanical power (J/min)	23.5 (10.6)	22.4 (9.3)	25.5 (12.4)	< 0.001
NMBs, n (%)	29 (4.2)	19 (4.3)	10 (4.0)	1.000
Prone position, n (%)	128 (18.1)	68 (14.9)	60 (23.8)	0.004
RM, n (%)	40 (5.6)	21 (4.6)	19 (7.5)	0.145
Hemodynamic parameters				
Heart rate (bpm)	100 [96, 102]	100 [88, 101]	100 [98, 111]	0.001
MAP (mmHg)	85.0 (10.1)	86.2 (10.0)	83.0 (10.1)	< 0.001
Vasopressor, n (%)	514 (72.5)	304 (66.5)	210 (83.3)	< 0.001
Lactate (mmol/L)	1.8 [1.2, 2.9]	1.7 [1.1, 2.6]	2.1 [1.5, 3.6]	< 0.001

Crs: Respiratory system compliance; DP: driving pressure; FiO_2_: fraction of inspired oxygen; PaO_2_: Partial arterial oxygen pressure; PaCO_2_: partial arterial carbon dioxide pressure; NMBs: neuromuscular blockers; PBW: predicted body weight; PEEP: positive end-expiratory pressure; Ppeak: peak inspiratory pressure; TV: tidal volume; RM: recruitment maneuver; MAP: mean arterial pressure. Data are presented as mean (SD), median (interquartile range [IQR]), or counts (proportion)

A total of 252 (35.5%) patients died within 28 days after invasive mechanical ventilation. ICU and hospital mortality rates were 26.0% and 27.9%, respectively (Table 1). The overall 28-day mortality was the highest in the hypercapnic patients, followed by the hypocapnic patients, and was the lowest in the normocapnic patients (Fig. 1).

## Association between time-varying PaCO_2_ and 28-day mortality in ARDS patients

After adjusting for age, APACHE II score, PaO_2_/FiO_2_, respiratory rate, tidal volume, ventilatory ratio, PEEP, dynamic driving pressure, and mechanical power, both time-varying PaCO_2_ (HR 1.07, 95% CI 1.03–1.11, p < 0.001) and time-varying CV-PaCO_2_ (HR 1.24 per 10% increase, 95% CI 1.10–1.40, p < 0.001) were independently associated with an increased hazard of 28-day mortality (Table 3). Additionally, a higher APACHE II score at baseline was independently associated with increased 28-day mortality. The results were largely consistent across sensitivity analysis including patients who still received invasive mechanical ventilation on Day 5 (Table S3).

**Table 3:** Multivariate Cox regression assessing the association of time-varying PaCO_2_ with 28-day mortality

	Time-varying PaCO_2_		Time-varying CV for PaCO_2_	
	Hazards ratio (95%CI)	P values	Hazards ratio (95%CI)	P values
Baseline variables				
Age, years	1.00 (0.99–1.01)	0.708	1.02 (1.01–1.03)	< 0.001
APACHE II score	1.05 (1.03–1.07)	< 0.001	1.06 (1.04–1.07)	< 0.001
PaO_2_/FiO_2_, per 10mmHg	1.18 (0.87–1.60)	0.288	0.93 (0.66–1.30)	0.664
Time-varying variables				
Respiratory rate, bpm	1.06 (1.00-1.13)	0.035	1.02 (0.99–1.06)	0.185
Tidal volume, mL/kg PBW	1.18 (0.98–1.42)	0.080	0.93 (0.83–1.05)	0.232
Ventilatory ratio	0.99 (0.81–1.21)	0.914	0.99 (0.70–1.38)	0.938
PEEP, cmH_2_O	1.05 (0.99–1.11)	0.085	1.15 (1.09–1.20)	< 0.001
Driving pressure, cmH_2_O	0.99 (0.94–1.04)	0.557	1.01 (0.97–1.05)	0.591
Mechanical power, J/min	1.04 (1.00-1.08)	0.072	1.02 (0.99–1.05)	0.245
PaCO_2_, mmHg	1.07 (1.03–1.11)	< 0.001	-	-
CV-PaCO_2_, per 10%	-	-	1.24 (1.10–1.40)	< 0.001

APACHE II: Acute Physiology and Chronic Health Evaluation II; PBW: predicted body weight; FiO_2_: fraction of inspired oxygen; PaO_2_: Partial arterial oxygen pressure; PaCO_2_: partial arterial carbon dioxide pressure; PEEP: positive end-expiratory pressure; CV: coefficient of variation; CI: confidence interval

**Fig. 2:**
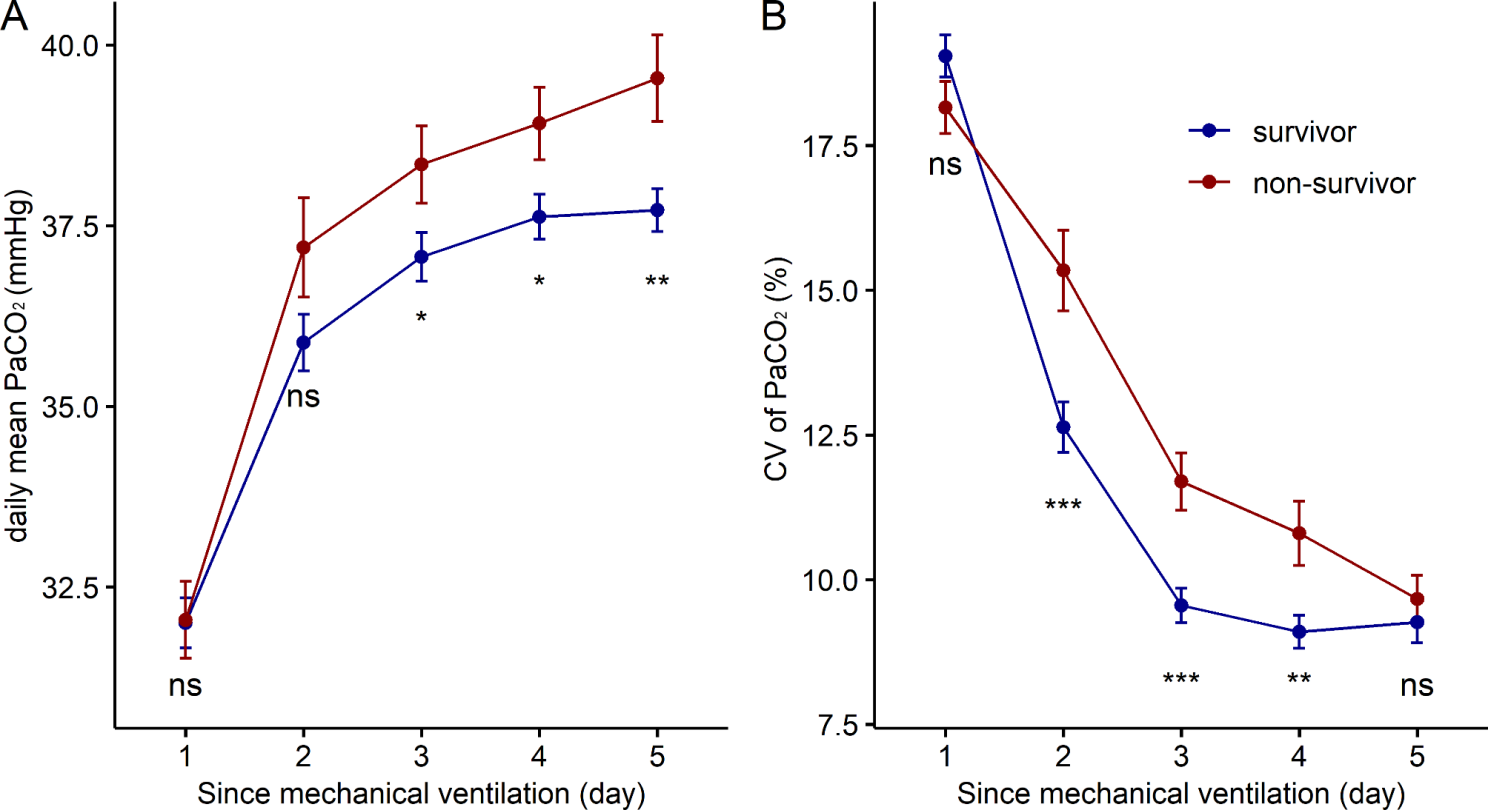
Comparisons of time-varying PaCO_2_ over time between 28-day survivors and non-survivors at the same time point. **A**: daily mean PaCO_2_. **B**: daily coefficient of variation for PaCO_2_. ^*^P < 0.05, ^**^P < 0.01, ^***^P < 0.001

The longitudinal values of the mean daily PaCO_2_ and coefficient of variation for PaCO_2_ over the first five days after mechanical ventilation are shown in Fig. 2. There was no significant difference in the mean PaCO_2_ or CV-PaCO_2_ between survivors and non-survivors on the first day of mechanical ventilation. PaCO_2_ gradually increased over time in both groups and was significantly lower in the 28-day survivors than in non-survivors after Day 3 (Fig. 2A). The CV-PaCO_2_ showed a decreasing tendency in all patients, which meant that the PaCO_2_ gradually stabilized. Compared with the survivors, the daily CV-PaCO_2_ was higher in the non-survivors for most of the time (Fig. 2B).

**Fig. 3:**
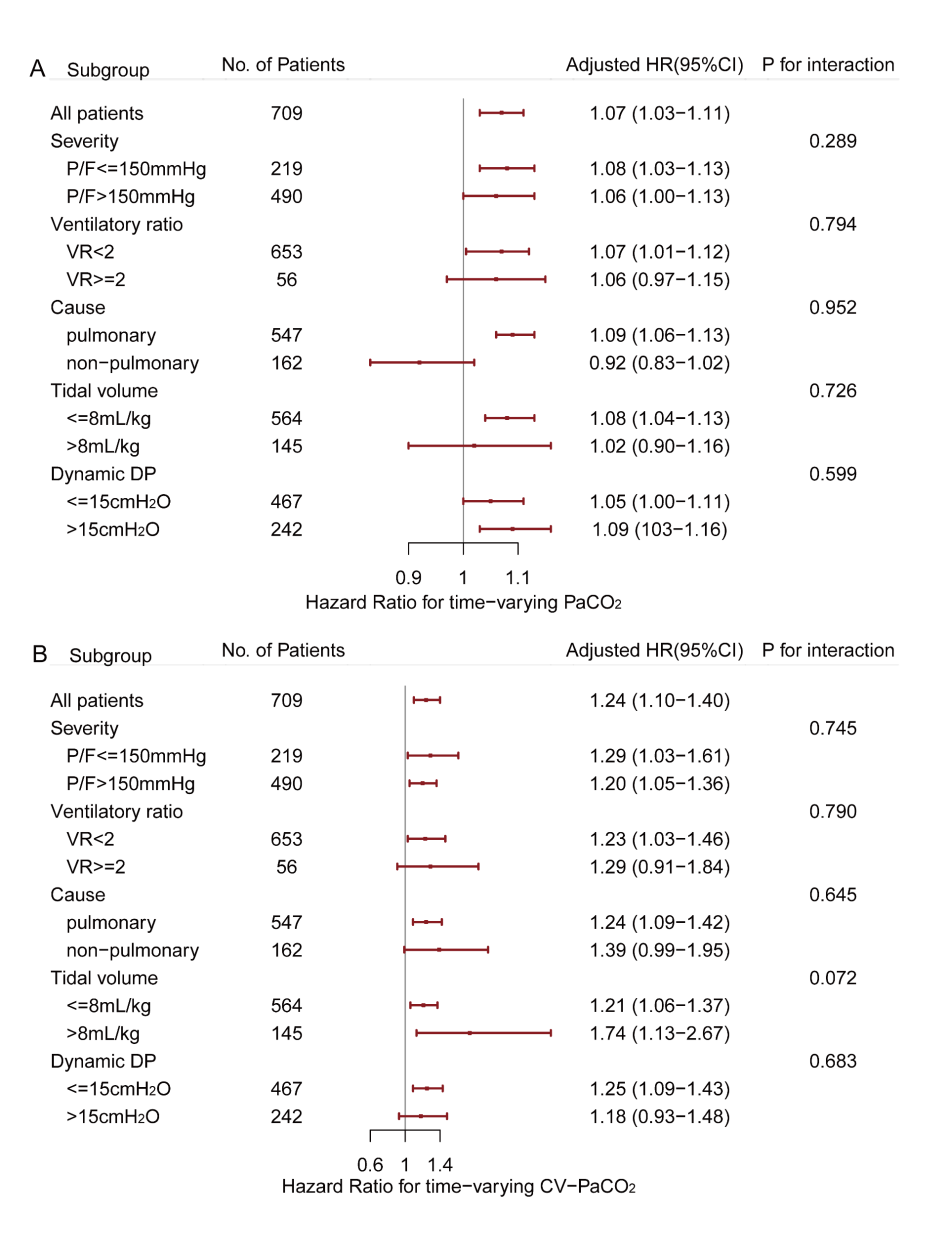
Subgroup analyses of the association between **A**: time-varying PaCO_2_. **B**: the coefficient of variation for PaCO_2_ with 28-day mortality

The results of the subgroup analyses of the association between time-varying PaCO_2_ and CV-PaCO_2_ with 28-day mortality are shown in Fig. 3. The association seemed to be stronger in patients exhibiting pulmonary ARDS, presenting a low ventilatory ratio (VR < 2), and receiving lung-protective ventilation (tidal volume ≤ 8 mL/kg PBW and dynamic driving pressure ≤ 15 cmH_2_O), and no interaction was detected.

## Cumulative effect of PaCO_2_ on 28-day mortality in ARDS patients

After adjustment for confounders as mentioned above, a higher proportion of normocapnia among all arterial blood gas analyses (HR 0.72 per 10% increase, 95% CI 0.58–0.89, p = 0.002) during the first five days since invasive mechanical ventilation was independently associated with a decreased hazard of death on Day 28 (Table S4). Consistently, the cumulative proportion of exposure to abnormal PaCO_2_ showed a decreased relationship with 28-day survival (Fig. S1). Similarly, exposure to PaCO_2_ dysregulation was generally associated with reduced hospital and ICU survival rates as well as shorter VFDs by Day 28 (Fig. S1).

## Discussion

These data highlighted the importance of CO_2_ management in mechanically ventilated ARDS patients. Time-varying PaCO_2_, measured by daily PaCO_2_ and the coefficient of variation for PaCO_2_ during the early phase of mechanical ventilation, independently influenced the clinical outcomes. Additionally, we observed a cumulative effect of PaCO_2_ derangement over time: every additional exposure to abnormal PaCO_2_ was associated with an increased risk of death. Therefore, limiting exposure to hypocapnia and hypercapnia in ARDS patients could improve outcomes.

The association between PaCO_2_ and clinical outcomes has solid biological plausibility. PaCO_2_ can be significantly influenced by disease severity and clinical management and has profound pathophysiological effects. PaCO_2_ may increase due to alveolar hypoventilation, resulting from increased dead space and massive shunt fraction or low tidal volume ventilation. Either the increased dead space [17] responsible for hypercapnia, or the hemodynamic instability rendered by hypercapnia [18, 19] is associated with poor outcomes. An important tactic to compensate for gas exchange is to increase cardiac output and subsequently altered lung perfusion [20]. The PaCO_2_ derangement in the non-survivors may due to hemodynamic impairment. Although studies have found that hypercapnia can alleviate the inflammatory response [21], it may simultaneously inhibit innate immunity and suppress the clearance of pathogens [22, 23].

On the other hand, hypocapnia may occur when high respiratory drive and strong inspiratory efforts or unnecessary high tidal volume ventilation lead to alveolar hyperventilation with a dangerous increase of mechanical power. The high-stretch ventilation caused by patient self-induced lung injury [24] and ventilator-induced lung injury [25] may exacerbate the detrimental effects of hypocapnia. Preclinical studies have revealed that hypocapnia can inhibit the secretion of alveolar surfactant [26] and impair the hypoxic contraction of pulmonary vessels, consequently reducing lung compliance and aggravating the ventilation-perfusion mismatch [27]. To date, there is considerable variability in CO_2_ management.

Our findings that PaCO_2_ derangement is associated with an increased mortality rate are consistent with several prior studies [5, 6, 28], although the protocols are diverse. The main difference between the present study and prior studies is the assessment period of PaCO_2_. Published studies regarding the association between PaCO_2_ and outcomes are mainly limited to cross-sectional analyses of single-time point data. However, a single measurement of PaCO_2_ cannot precisely describe the overall PaCO_2_ during the whole disease course and, therefore, may not necessarily significantly affect mortality. In fact, our study has shown that PaCO_2_ varies during the early phase of illness. PaCO_2_ reflects the balance between carbon dioxide production and elimination, and there is no doubt that derangement in PaCO_2_ may pose high risks for unfavorable outcomes [29]. Our study can be considered a complement and refinement of the previous results by investigating the cumulative effect of abnormal PaCO_2_ on 28-day mortality.

The importance of dynamic variations in respiratory parameters has been exemplified by longitudinal comparisons of ventilatory ratio and mechanical power in COVID-19 ARDS patients [30, 31]. However, limited data are available regarding the effect of time-varying PaCO_2_. We focused on longitudinal PaCO_2,_ and the results were generally consistent with prior studies concerning dynamic PaCO_2_. Tiruvoipati and his colleagues [32] found that in patients with sepsis who received mechanical ventilation, CV-PaCO_2_ showed a persistent association with an increased odds ratio for mortality, likely reflecting physiological instability. Similarly, an early rapid change in PaCO_2_ after ECMO in patients with respiratory failure was correlated with an increased risk of neurological complications [33]. Regrettably, none of the above studies investigated the effects of daily PaCO_2_ in ARDS patients and failed to represent the frequency and persistence of abnormal carbon dioxide exposure. By accounting for baseline confounders and time-dependent PaCO_2_, we have confirmed the importance of repeated PaCO_2_ measurements across the entire course or at least during the early phase of mechanical ventilation in ARDS patients.

Our study has certain implications for the clinical practice of critically ill patients. Clinicians should pay close attention to PaCO_2_ from the start of mechanical ventilation in ARDS patients. Efforts should be made to prevent dramatic changes in PaCO_2_ and limit exposure to potentially harmful hypocapnia and hypercapnia. In addition to limiting the tidal volume and driving pressure, it is also crucial to ensure adequate alveolar ventilation to facilitate the stabilization of carbon dioxide removal, as indicated by the results of the subgroup analysis performed in the subpopulation that received lung protective ventilation. Rescue therapies such as extracorporeal carbon dioxide removal and ECMO should be initialized when severe hypercapnia persists despite optimal medical management [34, 35]. Moreover, numerous studies have illustrated the association between PaCO_2_ and clinical outcomes in a large population of non-ARDS patients receiving mechanical ventilation [36, 37]. We speculate that the time-varying PaCO_2_ may affect the prognosis in non-ARDS patients equally.

To our knowledge, this study is the first to evaluate the effects of time-varying PaCO_2_ and CV-PaCO_2_ in ARDS patients and highlight the importance of the cumulative effect of normocapnia. There are several limitations we should acknowledge. First, patients were retrospectively enrolled from a single center which may impede the generalization of the results. Of concern, our cohort is comparable with a national cross-sectional survey of ARDS patients with regard to baseline characteristics [1]. Even though we excluded 92 patients who received ECMO, several studies have found that in patients receiving ECMO, PaCO_2_ derangements, as well as large relative changes in PaCO_2,_ were associated with poor prognosis, supporting our results [32, 38]. Second, this observational study could not lead to causal inferences for any associations. Third, the frequency of PaCO_2_ measurements differs among patients, depending on the severity of the disease. It was perhaps impossible to accurately estimate the cumulative time that patients were exposed to abnormal PaCO_2_. Calculating the ratio of the number of normocapnic cases to the total number of tests may be a reasonable method to quantify the cumulative effect. Fourth, due to the limited number of enrolled patients and insufficient data on PaCO_2_ measurements, we only included the first five days of mechanical ventilation. We may have overlooked the impact of time-varying PaCO_2_ on clinical outcomes in the subsequent course of the illness. Nevertheless, epidemiological studies have found that the median duration of ventilation for ARDS patients was 6 to 8 days [1, 2]. Thus, the analysis of longitudinal PaCO_2_ for five days still makes sense. Finally, since PaCO_2_ variation over time could not comprehensively describe the complexity of ARDS severity and evolution, in addition that modifications of ventilatory parameters may make a difference in prognosis, there remains a paucity of prospective data and evidence to interpret the effect of those time-varying variables.

## Conclusion

In conclusion, PaCO_2_ should be carefully monitored in ARDS patients, especially during the early course of mechanical ventilation. Substantial variations in PaCO_2_ and cumulative exposure to PaCO_2_ derangements were found in this study to be independently associated with an increase in 28-day mortality. Prospective studies may further clarify the effects of PaCO_2_ and optimize the early management of CO_2_.

## Electronic supplementary material

Below is the link to the electronic supplementary material.

Supplementary Material 1

## Data Availability

The dataset used during the current study are available from the corresponding author on reasonable request.

## Abbreviations

APACHE II: Acute Physiology and Chronic Health Evaluation II
ICU: intensive care unit
FiO_2_: fraction of inspired oxygen
PaO_2_: Partial arterial oxygen pressure
PaCO_2_: partial arterial carbon dioxide pressure
PEEP: positive end-expiratory pressure
CV: coefficient of variation
PBW: predicted body weight
ICU: intensive care unit
LOS: length of stay
VFD: ventilator-free day
