# Supplementary material for: Association between the time-varying arterial carbon dioxide pressure and 28-day mortality in mechanically ventilated patients with acute respiratory distress syndrome
Source: BMC Pulm Med. 2023 Apr 19;23:129. doi: 10.1186/s12890-023-02431-6 (PMC10113995; doi:10.1186/s12890-023-02431-6)
Supplement: Supplementary file 1 — Supplementary Material 1 [file 12890_2023_2431_MOESM1_ESM.docx]

## Online supplement

**Data definition**

Several variables were calculated as follows: predicted body weight (PBW) was calculated as [50+0.91× (centimeters of height‒152.4)] in males and [45.5+0.91 (centimeters of height‒152.4)] in females. The ventilatory ratio was calculated [[1](#_ENREF_1_1)]{Sinha, 2019 #520} as [VE×PaCO_2_ /[PBW ×100×37.5], where VE is the minute ventilation expressed in mL/min. The driving pressure was calculated as Pplat minus PEEP. Respiratory system compliance was calculated as tidal volume divided by Pplat. Mechanical power was computed [[2](#_ENREF_1_2)] as [0.098×RR×Vt×(Ppeak‒0.5×(Pplat‒PEEP))] in volume-controlled ventilation, and [0.098×RR×Vt×(PEEP+ΔPinsp)] in pressure-controlled ventilation, where RR is the respiratory rate, and Vt is the tidal volume in liter, ΔPinsp is the inspiratory pressure. If data of Pplat was censored, Ppeak was considered equal to Pplat in pressure-regulated modes. The coefficient of variation for PaCO_2_ (CV-PaCO_2_) was calculated as the percentage of standard deviation to the mean PaCO_2_ over a specific period. Briefly, the daily CV-PaCO_2_ was calculated as the daily standard deviation to the daily mean PaCO_2_, which reflects the daily fluctuations of PaCO_2_.

**References**

1. Sinha P, Calfee CS, Beitler JR, Soni N, Ho K, Matthay MA, et al. Physiologic Analysis and Clinical Performance of the Ventilatory Ratio in Acute Respiratory Distress Syndrome. Am J Respir Crit Care Med. 2019;199(3):333-41.

2. Chiumello D, Gotti M, Guanziroli M, Formenti P, Umbrello M, Pasticci I, et al. Bedside calculation of mechanical power during volume- and pressure-controlled mechanical ventilation. Crit Care. 2020;24(1):417.

Table S1 Percentages of missing data in the variables of interest in the cohort

| Variables | Cohort (n=709) |
| --- | --- |
| Age (years) | 0% |
| BMI | 1.3% |
| APACHE II | 18.6% |
| SOFA | 37.9% |
| Temperature (℃) | 9.6% |
| Heart rate (bpm) | 4.5% |
| Respiratory rate (bpm) | 1.1% |
| MAP (mmHg) | 4.4% |
| pHa | 0% |
| PaCO_2_ (mmHg) | 0% |
| PaO_2_ (mmHg) | 0% |
| PaO_2_/FiO_2_ (mmHg) | 0% |
| Bicarbonate (mmol/L) | 0% |
| Lactate (mmol/L) | 0.1% |
| WBC count (×10^9^/L) | 0% |
| Hemoglobin (g/L) | 0% |
| Platelet count(×10^9^/L) | 0% |
| Creatinine (μmol/L) | 0% |
| Bilirubin (μmol/L) | 0% |
| Tidal volume (mL) | 2.1% |
| Tidal volume (mL/kg PBW) | 3.4% |
| Minute ventilation (L) | 2.1% |
| Ppeak (cmH_2_O) | 11.4% |
| PEEP (cmH_2_O) | 15.5% |
| Dynamic DP (cmH_2_O) | 23.0% |
| Dynamic Crs (mL/cmH_2_O) | 24.1% |
| Mechanical power (J/min) | 11.6% |
| Ventilatory ratio | 3.4% |

APACHE Ⅱ: Acute Physiology and Chronic Health Evaluation Ⅱ; ARDS: acute respiratory distress syndrome; BMI: body mass index; SOFA: Sequential Organ Failure Assessment; MAP: mean arterial pressure; Crs: Respiratory system compliance; DP: driving pressure; FiO_2_: fraction of inspired oxygen; PaO_2_: Partial arterial oxygen pressure; PaCO_2_: partial arterial carbon dioxide pressure; PBW: predicted body weight; PEEP: positive end-expiratory pressure; Ppeak: peak inspiratory pressure

Table S2 Clinical characteristics of enrolled patients.

|  | Overall (n=709) | Survivors (n=457) | Non-survivors (n=252) | P value |
| --- | --- | --- | --- | --- |
| Laboratory tests |  |  |  |  |
| WBC count(×10^9^/L) | 12.7 (8.7) | 12.4 (7.4) | 13.2 (10.6) | 0.196 |
| Hemoglobin (g/L) | 104.4 (26.4) | 105.6 (26.4) | 102.3 (26.4) | 0.118 |
| Platelet count(×10^9^/L) | 160.0 (110.8) | 162.6 (107.8) | 155.2 (116.2) | 0.393 |
| C-reactive protein (mg/L) | 120.0 [55.3, 191.5] | 113.0 [42.6, 190.2] | 125.0 [80.9, 195.5] | 0.019 |
| Procalcitonin (ng/mL) | 2.3 [0.5, 9.2] | 2.2 [0.4, 9.4] | 2.7 [0.5, 8.9] | 0.333 |
| Creatinine (μmol/L) | 103.0 [70.0, 166.0] | 96.0 [68.0, 149.0] | 114.5 [77.0, 199.0] | <0.001 |
| Bilirubin (μmol/L) | 11.4 [6.8, 21.5] | 11.8 [7.3, 21.5] | 10.7 [5.3, 21.4] | 0.139 |

ARDS: acute respiratory distress syndrome; SOFA: sequential organ failure assessment; MAP: mean arterial pressure; WBC: white blood cell; Data are presented as mean (SD), median (interquartile range [IQR]), or counts (proportion).

Table S3 Multivariate Cox regression assessing the association of time-varying PaCO_2_ with 28-day mortality among patients with mechanical ventilation on Day 5 (n=474).

|  | Time-varying PaCO_2_ | | Time-varying CV for PaCO_2_ | | |  |
| --- | --- | --- | --- | --- | --- | --- |
|  | Hazards ratio (95%CI) | P values | | Hazards ratio (95%CI) | P values | |
| Baseline variables |  |  | |  |  | |
| Age, years | 1.00 (0.98-1.01) | 0.406 | | 1.02 (1.01-1.03) | <0.001 | |
| APACHE Ⅱ score | 1.04 (1.01-1.06) | 0.003 | | 1.04 (1.02-1.06) | <0.001 | |
| PaO_2_/FiO_2_, per 10mmHg | 0.93 (0.60-1.44) | 0.747 | | 1.05 (0.69-1.59) | 0.832 | |
| Time-varying variables |  |  | |  |  | |
| Respiratory rate, bpm | 1.08 (1.01-1.16) | 0.026 | | 1.03 (0.99-1.07) | 0.173 | |
| Tidal volume, mL/kg PBW | 1.32 (1.05-1.67) | 0.017 | | 0.99 (0.86-1.15) | 0.943 | |
| Ventilatory ratio | 1.03 (0.84-1.25) | 0.691 | | 0.97 (0.60-1.56) | 0.900 | |
| PEEP, cmH_2_O | 1.05 (0.99-1.11) | 0.639 | | 1.12 (1.05-1.18) | <0.001 | |
| Driving pressure, cmH_2_O | 0.99 (0.94-1.04) | 0.133 | | 1.00 (0.96-1.05) | 0.856 | |
| Mechanical power, J/min | 1.04 (1.00-1.08) | 0.202 | | 1.00 (0.96-1.05) | 0.864 | |
| PaCO_2_, mmHg | 1.07 (1.03-1.11) | <0.001 | | - | - | |
| CV-PaCO_2_, per 10% | - | - | | 1.22 (1.07-1.40) | 0.003 | |

Table S4 Cox regression assessing the cumulative effect of PaCO_2_ on 28-day mortality

| Variables | Adjusted HR | 95% CI | P value |
| --- | --- | --- | --- |
| Age, years | 1.02 | 1.01-1.03 | <0.001 |
| APACHE Ⅱ | 1.03 | 1.02-1.05 | <0.001 |
| PaO_2_/FiO_2_, per 10mmHg | 0.81 | 0.58-1.12 | 0.203 |
| Respiratory rate, bpm | 1.01 | 0.98-1.04 | 0.614 |
| Ventilatory ratio | 1.10 | 0.88-1.37 | 0.412 |
| Tidal volume, mL/kg PBW | 0.94 | 0.86-1.03 | 0.194 |
| PEEP, cmH_2_O | 1.06 | 1.02-1.10 | 0.006 |
| Driving pressure, cmH_2_O | 1.03 | 1.00-1.06 | 0.051 |
| Mechanical power, J/min | 1.00 | 0.99-1.02 | 0.622 |
| Proportion of normal PaCO_2，_per 10% | 0.72 | 0.58-0.89 | 0.002 |

APACHE Ⅱ: Acute Physiology and Chronic Health Evaluation Ⅱ; PBW: predicted body weight; FiO_2_: fraction of inspired oxygen; PaO_2_: Partial arterial oxygen pressure; PaCO_2_: partial arterial carbon dioxide pressure; HR: hazard ratio; CI: confidence interval.


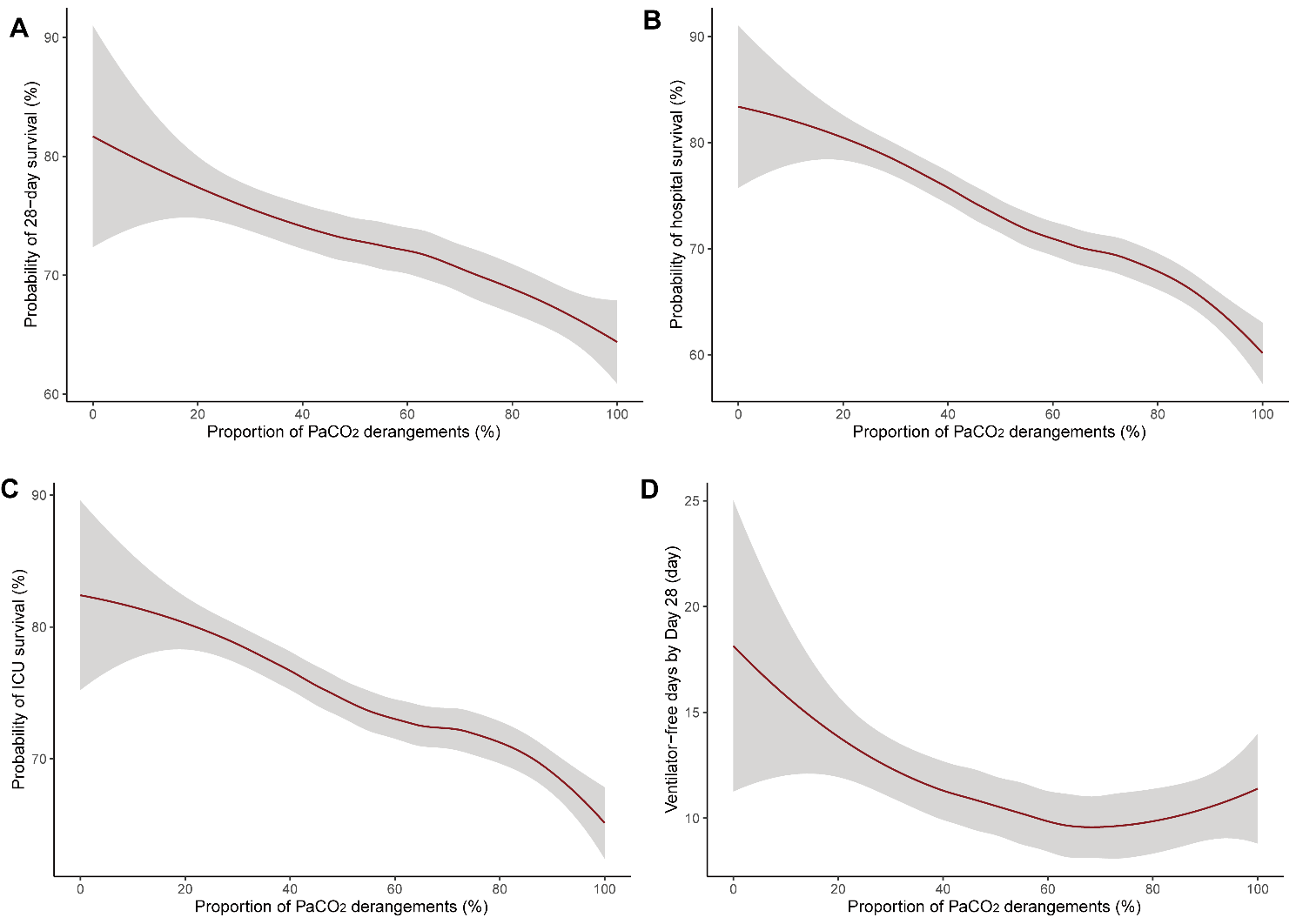


Figure S1. Association between proportion of PaCO_2_ derangements and clinical outcomes. A: probability of 28-day survival; B: probability of hospital survival; C: probability of ICU survival; D: ventilator-free days by Day 28. The line represents the predicted value and the gray area represents 95% confidence interval.
